# Comprehensive Identification of PTI Suppressors in Type III Effector Repertoire Reveals that *Ralstonia solanacearum* Activates Jasmonate Signaling at Two Different Steps

**DOI:** 10.3390/ijms20235992

**Published:** 2019-11-28

**Authors:** Masahito Nakano, Takafumi Mukaihara

**Affiliations:** 1 Research Institute for Biological Sciences, Okayama (RIBS), 7549-1 Yoshikawa, Kibichuo-cho, Okayama 716-1241, Japan; 2 Graduate School of Environmental and Life Science, Okayama University, 1-1-1 Tsushima-naka, Kita-ku, Okayama 700-8530, Japan

**Keywords:** *Ralstonia solanacearum*, type III effector, jasmonic acid, salicylic acid, *Nicotiana* plants

## Abstract

*Ralstonia solanacearum* is the causative agent of bacterial wilt in many plants. To identify *R. solanacearum* effectors that suppress pattern-triggered immunity (PTI) in plants, we transiently expressed *R. solanacearum* RS1000 effectors in *Nicotiana benthamiana* leaves and evaluated their ability to suppress the production of reactive oxygen species (ROS) triggered by flg22. Out of the 61 effectors tested, 11 strongly and five moderately suppressed the flg22-triggered ROS burst. Among them, RipE1 shared homology with the *Pseudomonas syringae* cysteine protease effector HopX1. By yeast two-hybrid screening, we identified jasmonate-ZIM-domain (JAZ) proteins, which are transcriptional repressors of the jasmonic acid (JA) signaling pathway in plants, as RipE1 interactors. RipE1 promoted the degradation of JAZ repressors and induced the expressions of JA-responsive genes in a cysteine–protease-activity-dependent manner. Simultaneously, RipE1, similarly to the previously identified JA-producing effector RipAL, decreased the expression level of the salicylic acid synthesis gene that is required for the defense responses against *R. solanacearum*. The undecuple mutant that lacks 11 effectors with a strong PTI suppression activity showed reduced growth of *R. solanacearum* in *Nicotiana* plants. These results indicate that *R. solanacearum* subverts plant PTI responses using multiple effectors and manipulates JA signaling at two different steps to promote infection.

## 1. Introduction

Plants are exposed to various abiotic and biotic stresses during their life cycle. To combat pathogens, plants have developed a specialized surveillance system, the so-called pattern-triggered immunity (PTI), to reject or attenuate infection by potential pathogens [1]. In PTI, plants sense evolutionarily conserved molecules from diverse pathogens, namely, pathogen/microbe-associated molecular patterns (PAMPs), such as flagellin, cold shock protein, and chitin, through pattern-recognition receptors (PRRs) on the plasma membrane [2]. The recognition of PAMPs by PRRs activates a large set of physiological responses including ion-flux changes, generation of reactive oxygen species (ROS), phosphorylation of mitogen-activated protein kinases, deposition of callose, production of phytohormones, and transcriptional reprogramming of defense-related genes, conferring disease resistance to a wide variety of pathogens.

Phytohormones act as signaling molecules that are required for immune responses against attacks from pathogens. Salicylic acid (SA) mediates defense responses against biotrophic and hemibiotrophic pathogens, whereas jasmonic acid (JA) controls defense responses against necrotrophic pathogens [3,4]. In many cases, their signaling network shows an antagonistic relationship with each other to induce appropriate immune responses against various pathogens with different infection strategies. During the coevolutionary arms race between pathogens and their host plants, pathogens acquired various virulence strategies to manipulate host hormonal signaling networks to accelerate successful infection [5]. One well-known example is the polyketide toxin coronatine (COR) produced by the hemibiotrophic bacterial pathogen *Pseudomonas syringae* pv. *tomato* (Pto) DC3000 [6]. COR is composed of two moieties, coronafacic acid and coronamic acid, and functions as a structural mimic of an active isoleucine conjugate of JA (JA-Ile). In the presence of COR, the F-box protein coronatie-insensitive1 (COI1) can promote the degradation of jasmonate-ZIM-domain (JAZ) proteins that repress the JA signaling pathway, resulting in the activation of JA signaling [7,8]. Upon Pto infection, the activation of JA signaling by COR antagonistically suppresses the SA-mediated signaling pathway, leading to the inhibition of stomatal closure and callose deposition to promote bacterial infection [9,10,11].

Many plant pathogenic bacteria have evolved a series of secretary proteins called effector proteins and inject them into plant cells via the Hrp type III secretion system to subvert plant immune responses [12]. Pathogen effectors often localize to specific organelles and exert their virulence functions in the early stage of infection. For example, AvrPtoB from Pto DC3000 degrades *Arabidopsis* PRR FLS2 through the E3 ubiquitin ligase activity to suppress PTI responses [13]. HopM1 localizes to endosomes and induces the proteasomal degradation of its target protein, AtMIN7, which is involved in PTI responses [14].

*Ralstonia solanacearum* is a Gram-negative phytopathogenic bacterium that causes bacterial wilt disease in more than 200 plant species, such as tomato, potato, banana, and eggplant [15]. The pathogen injects approximately 70 type III effectors into plant cells through the Hrp type III secretion system [16,17]. To date, several studies have clarified the biochemical functions of *R. solanacearum* effectors in PTI suppression. RipP2 suppresses the expressions of defense-related genes by acetylating WRKY transcription factors [18]. RipAY suppresses PTI by degrading glutathione in plant cells [19,20]. RipAR and RipAW suppress PTI responses through their E3 ubiquitin ligase activity [21]. RipAK inhibits the activity of host catalases and suppresses a hypersensitive response [22]. RipAL suppresses the SA signaling pathway by activating JA production in plant cells [23]. RipN suppresses PTI and alters the NADH/NAD^+^ ratio in plant cells through its ADP-ribose/NADH pyrophosphorylase activity [24]. However, the functions of other effectors are as yet largely unknown.

To expand our knowledge of *R. solanacearum* effectors in PTI suppression, in this study, we comprehensively screened for *R. solanacearum* RS1000 effectors with the ability to suppress flg22-triggered ROS burst in *N. benthamiana*. We identified 16 effectors that show PTI suppression activity. The detailed functional analysis of one of the effectors, RipE1, revealed that *R. solanacearum* manipulates the plant JA signaling pathway at two different steps to suppress SA-mediated defense responses. We also show that these PTI suppressors collectively contribute to bacterial virulence in *Nicotiana* plants.

## 2. Results

### 2.1. Identification of R. solanacearum Effectors that Suppress Flg22-Triggered ROS Burst in N. benthamiana

**Figure 1:**
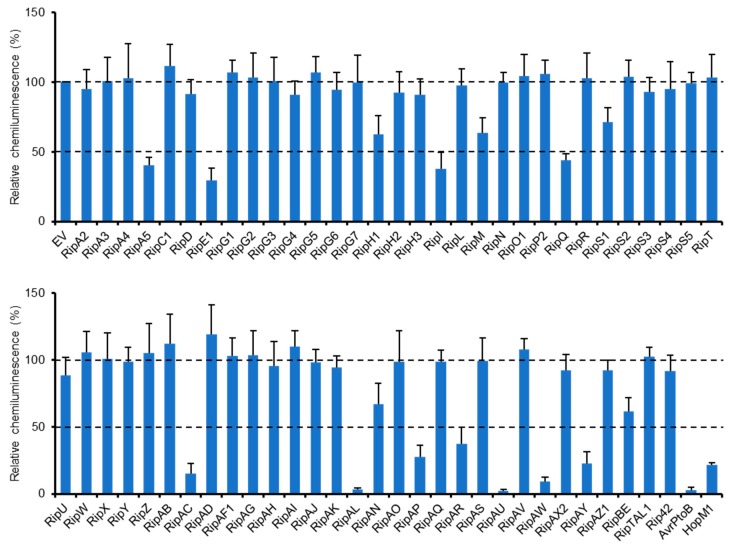
Identification of *R. solanacearum* effectors that suppress flg22-triggered ROS burst. Leaves of *N. benthamiana* were infiltrated with *A. tumefaciens* harboring the binary vector expressing the effector or empty vector (EV). Leaf disks were treated with the flg22 elicitor 2 days after agroinfiltration, and ROS production was monitored as photon counts for 120 min. Total photon counts are shown. Values are means ±SD of four replicates.

We previously identified a type III effector repertoire of *R. solanacearum* strain RS1000 [16] and constructed the binary vectors expressing effector genes under the control of a constitutive promoter [25]. To identify RS1000 effectors that affect plant PTI responses, we transiently expressed each effector protein in *N. benthamiana* leaves by agroinfiltration and evaluated its ability to suppress ROS burst triggered by flg22 treatment. Among the effector repertoire of RS1000, three effectors, namely, RipB, RipP1, and RipAA, were excluded from the screening because these effectors act as avirulence determinants and induce rapid effector-triggered immunity (ETI) responses in *N. benthamiana* [26]. In this screening, two type III effectors, AvrPtoB and HopM1, from Pto DC3000 with the ability to suppress PTI [13,14] were used as a positive control and an empty vector (EV) as a negative control. Out of the 61 Rip effectors tested, 11 (RipA5, RipE1, RipI, RipQ, RipAC, RipAL, RipAP, RipAR, RipAU, RipAW, and RipAY) strongly (≤50%) suppressed flg22-triggered ROS burst compared with the EV control (Figure 1). Among them, four effectors (RipAL, RipAR, RipAW, and RipAY) were previously shown to suppress plant PTI responses [19,21,23], indicating that our screening worked effectively. We also identified five effectors that weakly (51–70%) suppress ROS burst in *N. benthamiana*. By the screening, we identified a total of 16 effectors that affect flg22-triggered ROS burst.

### 2.2. RipE1 is a Member of the HopX Family and Suppresses PTI through its Cysteine Protease Activity

Among the seven newly identified effectors that strongly suppress flg22-triggered ROS burst, we focused on RipE1 because it showed sequence similarity to *P. syringae* effectors belonging to the HopX family, such as HopX1 from *P. syringae* pv. *tabaci* 11528 (HopX1_Pta_) (21% identity and 69% similarity; Figure S1). HopX1_Pta_ has been shown to activate plant JA signaling by directly degrading JAZ repressors with its cysteine protease activity [27]. Although the entire sequence homology between RipE1 and HopX1_Pta_ is low, the cysteine, histidine, and aspartic acid residues corresponding to the catalytic triad of HopX1_Pta_ were conserved in RipE1 as C172, H203, and D222, respectively (Figure S1). We previously showed that the *R. solanacearum* effector RipAL activates JA signaling by inducing JA production to suppress SA-mediated defense responses in plant cells [23]. It has been proposed that RipAL targets chloroplast lipids and releases JA precursors to induce JA production because RipAL contains a putative lipase domain similar to that of the *Arabidopsis* DAD1 lipase that is involved in JA biosynthesis through the production of JA precursors [28]. To elucidate whether RipE1 manipulates and activates JA signaling by a mechanism different from that used by RipAL, we tested the cysteine protease activity of RipE1. We transiently expressed hemagglutinin (HA)-tagged RipE1 in *N. benthamiana* leaves by agroinfiltration (Figure S2A) and purified the recombinant RipE1 proteins using an anti-HA affinity resin. A protease activity assay using the fluorescein-labeled casein substrate revealed that RipE1 showed a clear protease activity in vitro compared with the buffer control (Figure S3). We also constructed two RipE1 mutants, in which the cysteine at position 172 was changed to alanine (RipE1^C172A^), and the histidine at position 203 was also changed to alanine (RipE1^H203A^). The recombinant RipE1^C172A^ and RipE1^H203A^ proteins lost their protease activity in vitro. This finding indicates that RipE1 has cysteine protease activity and that the two putative catalytic residues are essential for its protease activity.

**Figure 2:**
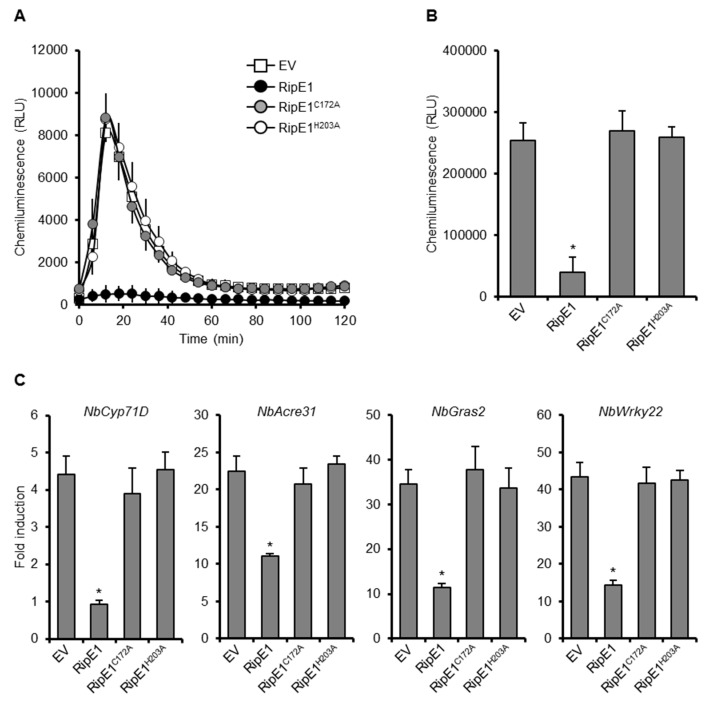
Effect of RipE1 expression on PTI responses in *N. benthamiana*. Leaves were infiltrated with *A. tumefaciens* harboring the binary vector expressing N-terminal HA-tagged RipE1, RipE1^C172A^, or RipE1^H203A^, or EV. (**A**) Flg22-triggered reactive oxygen species (ROS) burst in leaves expressing RipE1 and its catalytic site mutants. Leaf disks were treated with the flg22 elicitor 2 days after agroinfiltration, and ROS production was monitored as photon counts for 120 min. Values are means ±SD of six replicates. (**B**) Total photon counts in (**A**). The asterisk (*) denotes a statistically significant difference compared with the EV control (*p* < 0.01, Student’s *t*-test). (**C**) Expression levels of PTI marker genes in leaves expressing RipE1 and its mutants. Leaves were treated with the flg22 elicitor 2 days after agroinfiltration, and total RNA was isolated from leaves 60 min after the treatment. Expression levels were determined by qRT-PCR analysis and normalized to that in the water treatment. Values are means ±SD of three replicates. Asterisks (*) denote statistically significant differences compared with the EV control (*p* < 0.01, Student’s *t*-test). All experiments were repeated three times with similar results, and representative results are shown.

To clarify the role of the protease activity of RipE1 in PTI suppression, we transiently expressed HA-tagged RipE1, RipE1^C172A^, and RipE1^H203A^ in *N. benthamiana* leaves by agroinfiltration. The expression of RipE1, but not those of RipE1^C172A^ and RipE1^H203A^, suppressed the flg22-triggered ROS burst (Figure 2A,B) and the expressions of PTI marker genes (Figure 2C) compared with the EV control. We confirmed that the expression of RipE1 induced no visible changes in colors and ion leakage levels in the infiltrated leaves at least 3 days after agroinfiltration (Figure S2B,C). These findings show that RipE1 suppresses PTI through its cysteine protease activity.

### 2.3. RipE1 Localizes to Nucleocytoplasm in Plant Cells

**Figure 3:**
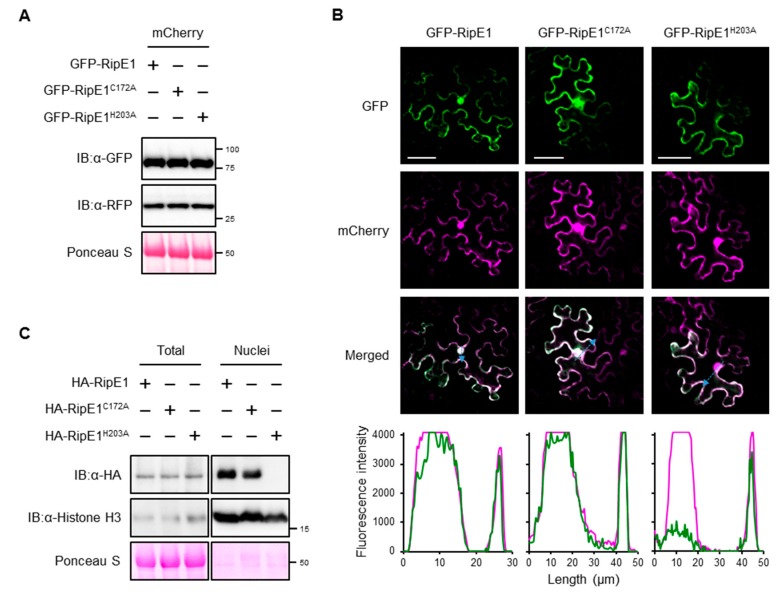
Subcellular localization of RipE1 in *N. benthamiana* cells. (**A**) Immunoblot analysis of green fluorescent protein (GFP)-RipE1 fusions. Leaves were coinfiltrated with *A. tumefaciens* harboring the binary vector expressing N-terminal GFP-tagged RipE1, RipE1^C172A^, or RipE1^H203A^ and the binary vector expressing mCherry under the control of the β-estradiol-inducible promoter. Leaves were treated with β-estradiol 1 day after agroinfiltration. Total protein was extracted from the leaves 1 day after the treatment and subjected to immunoblot analysis using an anti-GFP or anti-RFP antibody. The membrane was stained with Ponceau S as the loading control. (**B**) Subcellular localization of GFP-RipE1 and its mutants. Fluorescence was observed 1 day after the treatment by confocal microscopy. The overlay of fluorescence was monitored by scanning fluorescence intensities in the regions indicated by blue arrows. Bars, 50 μm. (**C**) Immunoblot analysis of RipE1 in the nuclear fraction. Leaves were infiltrated with *A. tumefaciens* harboring the binary vector expressing N-terminal HA-tagged RipE1, RipE1^C172A^, or RipE1^H203A^. Total and nuclear fractions were extracted from leaves 2 days after agroinfiltration and subjected to immunoblot analysis using an anti-HA or anti-histone H3 antibody. The membrane was stained with Ponceau S as the loading control.

To examine the subcellular localization of RipE1 in plant cells, we constructed binary vectors expressing green fluorescent protein (GFP)-tagged RipE1, RipE1^C172A^, and RipE1^H203A^ under the control of the β-estradiol-inducible promoter. When the GFP-RipE1 fusions were transiently coexpressed with the nucleocytoplasm marker mCherry in *N. benthamiana* leaves by agroinfiltration (Figure 3A), the fluorescence signals of GFP-RipE1, GFP-RipE1^C172A^, and GFP-RipE1^H203A^ were observed in the cytoplasm of mesophyll cells as well as that of mCherry (Figure 3B). On the other hand, line-scanning analysis revealed that the fluorescence intensity of GFP-RipE1 or GFP-RipE1^C172A^, but not GFP-RipE1^H203A^, completely overlapped with that of mCherry in the nucleus. To verify this finding, we isolated total and nuclear protein fractions from *N. benthamiana* leaves expressing HA-tagged RipE1, RipE1^C172A^, and RipE1^H203A^ and analyzed the accumulation levels of RipE1 and its mutants by immunoblotting. RipE1^H203A^ was detected in the total fraction, but not in the nuclear fraction, whereas RipE1 and RipE1^C172A^ were detected in both fractions (Figure 3C). These observations indicate that RipE1 localizes to the nucleocytoplasm of *N. benthamiana* cells and that the H203A mutation affects not only the protease activity but also the nuclear localization of RipE1.

### 2.4. RipE1 Interacts with JAZ Proteins in Yeast and Plant Cells

**Figure 4:**
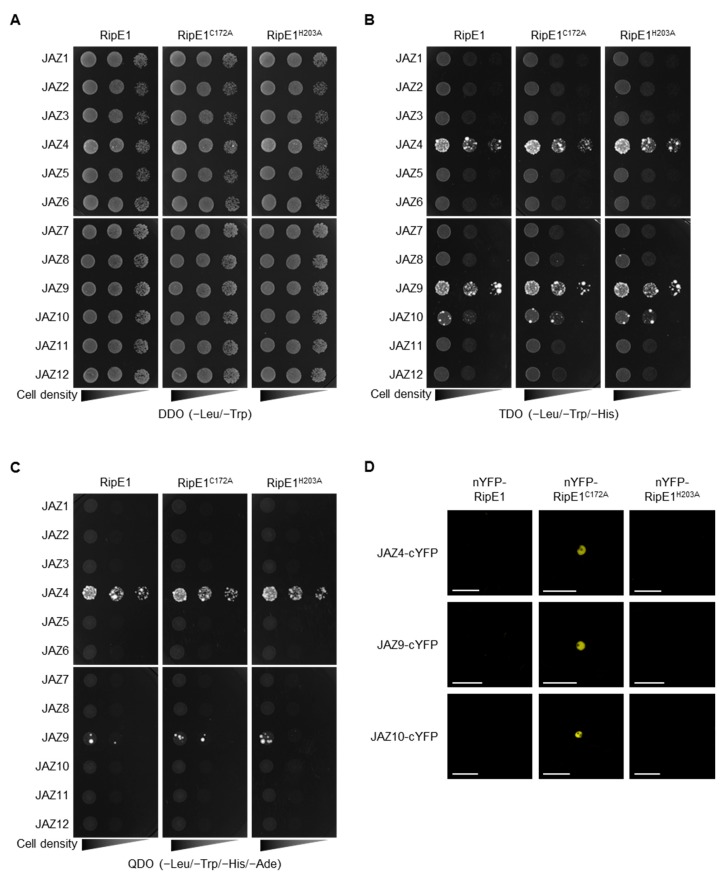
Interaction of RipE1 and jasmonate-ZIM-domain (JAZ) family. (**A**–**C**) Interaction of RipE1 and JAZ family in yeast. Serial dilutions of diploid cells harboring the bait vector expressing RipE1 and the prey vector expressing JAZs were spotted on DDO (**A**), TDO moderately selective (**B**), and QDO stringently selective (**C**) plates. Photographs were taken after 5 days of incubation. (**D**) Interaction of RipE1 and JAZ proteins in planta. Leaves of *N. benthamiana* were coinfiltrated with *A. tumefaciens* harboring the binary vector expressing N-terminal nYFP-tagged RipE1, RipE1^C172A^, or RipE1^H203A^ and the binary vector expressing C-terminal cYFP-tagged JAZ4, JAZ9, or JAZ10. Fluorescence was observed 2 days after agroinfiltration by confocal microscopy. Bars, 50 μm. All experiments were repeated three times with similar results, and representative results are shown.

To identify plant proteins that interact with RipE1, we performed yeast two-hybrid screening using RipE1 as the bait. Yeast cells expressing *ripE1* showed no significant difference in their growth (Figure 4A), indicating that RipE1 is not toxic to yeast cells. Upon screening approximately 2 × 10^6^ transformants with an *A. thaliana* cDNA-derived prey library, we identified JAZ4 as a candidate plant target of RipE1. The JAZ family consists of 12 members in *A. thaliana* [4,29]. We examined the interaction of RipE1 with the JAZ proteins and found that RipE1 interacts with JAZ4, JAZ9, and JAZ10 in yeast cells (Figure 4B), particularly strongly with JAZ4 and JAZ9 (Figure 4C). The interaction with JAZ proteins was not affected by the C172A and H203A mutations of RipE1 (Figure 4B,C).

JAZ proteins are mainly localized in the plant nucleus and act as repressors in the JA signaling pathway [4]. Indeed, fluorescence signals of GFP-tagged JAZ4, JAZ9, and JAZ10 were observed in the nucleus of *N. benthamiana* cells (Figure S4). To elucidate whether RipE1 interacts with JAZs in the plant nucleus, we performed a bimolecular fluorescence complementation (BiFC) assay using *N. benthamiana* leaves by agroinfiltration. Notably, when the catalytically inactive nYFP-RipE1^C172A^, but not the catalytically active nYFP-RipE1, was coexpressed with JAZ4-cYFP, JAZ9-cYFP, or JAZ10-cYFP, fluorescence signals were observed in the nucleus of *N. benthamiana* cells (Figure 4D). No fluorescence signal was observed when the catalytically inactive but non-nucleus-localized nYFP-RipE1^H203A^ was used in the assay. Collectively, these observations indicate that RipE1 interacts with JAZ4, JAZ9, and JAZ10, mainly in plant nuclei.

### 2.5. RipE1 Degrades JAZ Repressors to Activate JA Signaling and Simultaneously Suppresses SA Signaling

**Figure 5:**
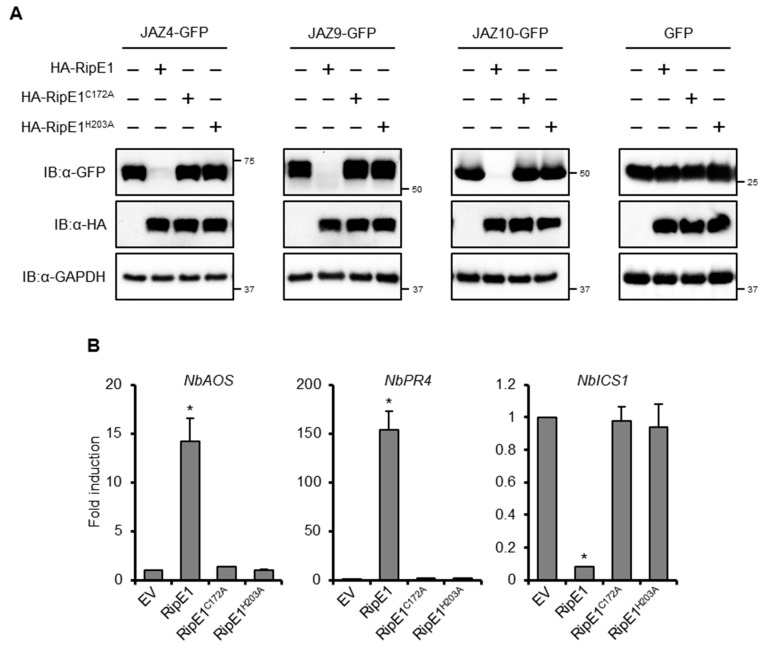
Effect of RipE1 expression on the accumulation of JAZs and defense-related gene expression in *N. benthamiana*. (**A**) Leaves were coinfiltrated with *A. tumefaciens* harboring the binary vector expressing N-terminal HA-tagged RipE1, RipE1^C172A^, or RipE1^H203A^ and the binary vector expressing C-terminal GFP-tagged JAZ4, JAZ9, JAZ10, or GFP alone. Total protein was extracted from the leaves 2 days after agroinfiltration and subjected to immunoblot analysis using an anti-HA or anti-GFP antibody. The membrane was subjected to immunoblot analysis using an anti-GAPDH antibody as the endogenous and loading control. (**B**) Expression levels of jasmonic acid (JA) and salicylic acid (SA) marker genes in leaves expressing RipE1 and its mutants. Leaves were infiltrated with *A. tumefaciens* harboring the binary vector expressing N-terminal HA-tagged RipE1, RipE1^C172A^, or RipE1^H203A^, or EV. Total RNA was isolated from leaves 2 days after agroinfiltration. Expression levels were determined by qRT-PCR analysis and normalized to that of EV. Values are means ±SD of three replicates. Asterisks (*) denote statistically significant differences compared with the EV control (*p* < 0.01, Student’s *t*-test). All experiments were repeated three times with similar results, and representative results are shown.

In the aforementioned BiFC experiments, no fluorescence signal was detected in the leaves coexpressing nYFP-RipE1 and JAZs-cYFP fusions (Figure 4D). To test whether RipE1 degrades JAZ proteins, we transiently coexpressed HA-tagged RipE1 and GFP-tagged JAZs in *N. benthamiana* leaves by agroinfiltration. The expression of RipE1 markedly decreased the accumulation of JAZ4-GFP, JAZ9-GFP, and JAZ10-GFP in the infiltrated leaves (Figure 5A). On the other hand, the expression of RipE1^C172A^ and RipE1^H203A^ did not affect the accumulation of JAZs-GFP fusions. These results show that RipE1 degrades at least three JAZ repressors through its cysteine protease activity in plant cells.

We observed that the prolonged expression of HA-tagged RipE1, but not RipE1^C172A^ or RipE1^H203A^, induced leaf chlorosis, and a reduction in the chlorophyll content of the leaves (Figure S2D,E), which are hallmark events accompanied by the activation of JA signaling [30,31]. Notably, the transient expression of RipE1, but not RipE1^C172A^ or RipE1^H203A^, induced the expressions of JA signaling marker genes, such as *NbAOS* and *NbPR4,* in *N. benthamiana* (Figure 5B). In contrast to JA signaling, the transient expression of RipE1 greatly decreased the expression level of the SA signaling marker gene *NbICS1* encoding the SA-producing enzyme. This finding indicates that RipE1 activates JA signaling by degrading JAZ repressors and simultaneously suppresses the antagonistic SA signaling pathway in *N. benthamiana*.

### 2.6. RipE1 can Complement the Impaired Virulence Phenotype of the COR-Deficient Mutant of Pto in Arabidopsis Plants

**Figure 6:**
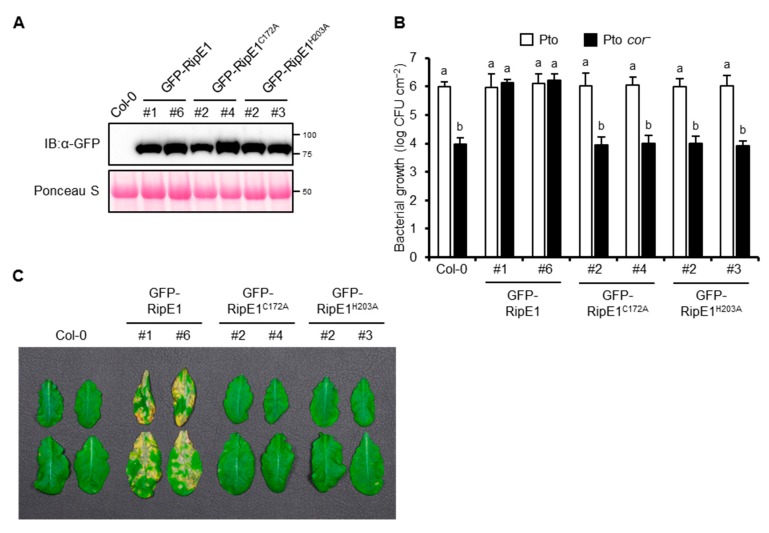
The phenotype of transgenic *A. thaliana* expressing RipE1. *A. thaliana* Col-0 and transgenic plants expressing N-terminal GFP-tagged RipE1, RipE1^C172A^, and RipE1^H203A^ under the control of the β-estradiol-inducible promoter were treated with β-estradiol. (**A**) Immunoblot analysis of GFP-RipE1 fusions. Total protein was extracted from the leaves 3 days after the treatment and subjected to immunoblot analysis using an anti-GFP antibody. The membrane was stained with Ponceau S as the loading control. (**B**) Growth of Pto and Pto *cor*^−^ in *A. thaliana* Col-0 and transgenic plants expressing GFP-RipE1 and its mutants. Leaves treated with β-estradiol for 1 day were sprayed with the bacterial suspension, and the bacterial population was determined 2 days after inoculation. Values are means ±SD of three replicates. Different letters denote statistically significant differences (*p* < 0.05, one-way ANOVA with Tukey–Kramer HSD test). (**C**) Disease symptoms caused by Pto *cor*^−^ in *A. thaliana* Col-0 and transgenic plants expressing GFP-RipE1 and its mutants. One day after treatment with β-estradiol, the leaves were sprayed with the bacterial suspension. Photographs were taken 1 week after inoculation. All experiments were repeated three times with similar results, and representative results are shown.

Pto DC3000, the causative agent of bacterial speck disease in tomato, could also infect *A. thaliana*. The *A. thaliana*–Pto interaction has been used as a model pathosystem for determining the contribution of effector proteins from other pathogens to virulence [21,24,32]. Therefore, we generated transgenic *A. thaliana* plants expressing GFP-RipE1, GFP-RipE1^C172A^, and GFP-RipE1^H203A^ under the control of the β-estradiol-inducible promoter (Figure 6A). The growth of Pto in the transgenic plant leaves expressing GFP-RipE1 showed no significant difference compared with that in the parental Col-0 leaves (Figure 6B). Pto produces the phytotoxin COR, which is a functional mimic of JA-Ile that activates JA signaling to suppress SA-mediated defense responses in plants [6,11]. The growth of the COR-deficient mutant of Pto (Pto *cor*^−^) decreased 100-fold compared with that of the wild-type strain in the Col-0 leaves (Figure 6B). Notably, the growth of the Pto *cor*^−^ mutant increased 100-fold and reached the wild-type level in the transgenic plants expressing GFP-RipE1, but not GFP-RipE1^C172A^ and GFP-RipE1^H203A^ compared with the parental Col-0 plants 2 days after inoculation. Moreover, the development of disease symptoms caused by Pto *cor*^−^ was accelerated in the transgenic plant leaves expressing GFP-RipE1, but not GFP-RipE1^C172A^ and GFP-RipE1^H203A^ (Figure 6C). These observations indicate that RipE1 can complement the impaired virulence phenotype of Pto *cor*^−^ through the activation of JA signaling.

### 2.7. Multiple Deletions of Effector Genes that Show a Strong PTI Suppression Activity Affect the Growth of R. solanacearum in Nicotiana Plants

**Figure 7:**
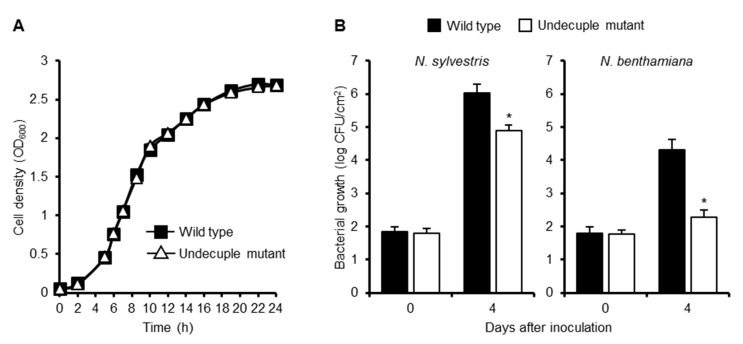
Contribution of PTI suppressors to virulence of *R. solanacearum* in *Nicotiana* plants. (**A**) Growth of *R. solanacearum* mutants in vitro. *R. solanacearum* wild-type strain and the Δ*ripA5* Δ*ripE1* Δ*ripI* Δ*ripQ* Δ*ripAC* Δ*ripAL* Δ*ripAP* Δ*ripAR* Δ*ripAU* Δ*ripAW* Δ*ripAY* undecuple mutant were diluted in BG medium to OD_600_ = 0.05. The cultures were incubated at 28 °C with shaking, and cell density was spectrometrically measured at the indicated time points. Values are means ±SD of three replicates. (**B**) Growth of *R. solanacearum* mutants *in planta*. Leaves of *N. sylvestris* and *N. benthamiana* were inoculated with the suspension of the wild type or undecuple mutant of *R. solanacearum*. The bacterial population was determined on the indicated days after inoculation. Values are means ±SD of four replicates. Asterisks (*) denote statistically significant differences compared with the wild type (*p* < 0.01, Student’s *t*-test). All experiments were repeated three times with similar results, and representative results are shown.

To evaluate the contribution of RipE1 to bacterial virulence, we generated a Δ*ripE1* mutant of *R. solanacearum* strain RS1002, a nalidixic acid-resistant derivative of RS1000, and inoculated the Δ*ripE1* mutant into solanaceous host plants. However, the Δ*ripE1* mutant showed no significant difference in symptom development in the inoculated plants compared with the wild-type strain (Figure S5), probably owing to functional redundancy among the effector repertoire. We next generated a Δ*ripA5* Δ*ripE1* Δ*ripI* Δ*ripQ* Δ*ripAC* Δ*ripAL* Δ*ripAP* Δ*ripAR* Δ*ripAU* Δ*ripAW* Δ*ripAY* undecuple mutant, in which all of the 11 effector genes that strongly suppressed flg22-triggered ROS burst in *N. benthamiana* were deleted. We monitored the growth of the wild-type strain and the undecuple mutant in vitro and *in planta*. Although the growth rates of the two strains showed no significant difference in a rich medium (Figure 7A), the undecuple mutant showed reduced bacterial growth in susceptible *N. sylvestris* leaves compared with the wild-type strain (Figure 7B). Furthermore, the undecuple mutant showed a significant growth defect in resistant *N. benthamiana* leaves. These findings indicate that the 11 effectors collectively contribute to the growth of *R. solanacearum* in *Nicotiana* plants.

## 3. Discussion

In this study, we screened *R. solanacearum* RS1000 effectors for their ability to suppress plant PTI responses using an *Agrobacterium*-mediated transient expression system. Out of the 61 effectors tested, 16 (26%) were found to suppress flg22-triggered ROS burst in *N. benthamiana* (Figure 1). Similar comprehensive screenings for bacterial effectors that suppress PTI have been performed in Pto and *Xanthomonas euvesicatoria*. In Pto, seven out of the 22 effectors tested (32%) can suppress flg22-triggered ROS burst and expression of PTI marker genes in *N. benthamiana* [33]. In *X. euvesicatoria*, 17 out of the 33 effectors tested (52%) show the ability to suppress flg22-triggered PTI signaling in *N. benthamiana* [34]. Our finding corresponds well to the previous studies revealing that many effectors in the effector repertoire of a plant pathogenic bacterium can suppress plant PTI responses.

Out of the 16 effectors identified by our screening, 11 strongly suppressed flg22-triggered ROS burst in *N. benthamiana* (Figure 1). Among them, RipE1 was found to share low homology with the cysteine protease effector HopX1_Pta_ from *P. syringae* pv. *tabaci* 11528 (Figure S1). We showed that RipE1 suppresses flg22-triggered ROS burst and reduces the expression levels of PTI marker genes in a protease-activity-dependent manner (Figure S3 and Figure 2). However, the functions of HopX family effectors vary among the members. For example, HopX1 from Pto DC3000 (HopX1_Pto_) does not show the protease activity in *N. benthamiana* [27]. For PTI suppression, HopX1_Pta_ and HopX1_Pto_ do not suppress flg22-induced ROS burst and expressions of PTI marker genes in *N. benthamiana* [33]. *X. euvesicatoria* possesses two HopX1 family effectors, XopE1 and XopE2 [35]. XopE2, but not XopE1, has been shown to suppress flg22-triggered PTI in *N. benthamiana,* although their enzymatic activity remains unclear [34]. In this study, we clearly demonstrated that cysteine protease activity is indispensable for the PTI suppression activity of RipE1.

For the function of RipE1 in plant cells, we obtained the following results. (i) RipE1 localized to the nucleocytoplasm of plant cells (Figure 3). (ii) RipE1 interacted with JAZ4, JAZ9, and JAZ10 in yeast and plant cells (Figure 4) and degraded the JAZ repressors in the nucleus when coexpressed in plant cells (Figure 4D and Figure 5A). (iii) RipE1 activated JA signaling and simultaneously suppressed SA signaling (Figure 5B). (iv) The expression of RipE1 complemented the reduced growth phenotype of the Pto *cor*^−^ mutant in *Arabidopsis* plants (Figure 6). It has been shown that HopX1_Pta_ degrades JAZ repressors through its cysteine protease activity and activates JA signaling to suppress SA signaling [27]. Except for the ability to suppress plant PTI responses, RipE1 functions similarly to HopX1_Pta_.

It is widely known that the JA and SA signaling pathways antagonize each other to fine-tune proper defense responses to combat against pathogens with different infection strategies. Upon activation of JA signaling, the MYC2 transcription factor released by the degradation of JAZ repressors induces the expression of NAC transcription factors that repress the expression of the SA synthesis enzyme ICS1, leading to the downregulation of SA signaling [11]. We showed that RipE1 activates JA signaling and simultaneously suppresses the expression of *NbICS1* in *N. benthamiana* (Figure 5). It has been shown that the virulence of *R. solanacearum* is enhanced in *NbICS1*-silenced plants [23]. Our work provides new evidence that *R. solanacearum* exploits antagonistic interactions between the SA and JA signaling pathways to suppress SA signaling in plants and promote successful infection. We previously showed that RipAL targets chloroplasts and induces JA production to activate JA signaling and simultaneously suppress SA-mediated defense responses in plants [23]. Our findings indicate that *R. solanacearum* uses at least two effectors that target different organelles and activate host JA signaling in two different steps, JA production and JAZ degradation, probably giving a synergistic effect (Figure S6).

We observed that RipE1 preferentially interacts with JAZ4, JAZ9, and JAZ10 among the JAZ members in the yeast two-hybrid system (Figure 4) and proteolytically degrades them in plant cells (Figure 5A). It has been reported that the loss-of-function mutant *jaz4-1* or *jaz10-1* in *A. thaliana* makes the plant hypersusceptible to Pto DC3000 and enhances pathogen growth in infected leaves compared with the wild-type plants [36,37]. No other single-gene mutations of 12 JAZ members affect the *in planta* growth of Pto DC3000, indicating that JAZ4 and JAZ10 play important roles in the defense response to the bacterial pathogen. It is noteworthy that RipE1 interacts most strongly with JAZ4 (Figure 4C) since the *jaz4-1* mutation showed the most striking effect in terms of the enhanced growth of the bacterial pathogen in mutant plants [37]. JAZ4 and JAZ9 belong to the same subgroup of JAZ members based on their amino acid sequences [29]; therefore, it is reasonable to consider that RipE1 interacts with JAZ9 (Figure 4). The roles of JAZ4, JAZ9, and JAZ10 in defense responses against *R. solanacearum* should be clarified in future work.

Generally, a single mutation in effector genes does not affect the virulence of plant pathogenic bacteria, probably owing to its functional redundancy among the effector repertoire. Therefore, we generated the undecuple mutant that lacks 11 effectors with a strong PTI suppression activity. Notably, the growth of the undecuple mutant was reduced in susceptible *N. sylvestris* leaves and extremely decreased in resistant *N. benthamiana* leaves (Figure 7). This finding clearly shows that Rip effectors with PTI suppression activity collectively promote the growth of *R. solanacearum* in plants. The greater inhibition of bacterial growth in resistant plants might be explained by the more rapid and stronger induction of defense responses in resistant plants. From another viewpoint, it has recently been suggested that one effector targets multiple plant factors to subvert immune responses. For example, the acetyltransferase effector HopZ1a from *P. syringae* degrades not only JAZ proteins but also GmHID1, which is involved in isoflavone biosynthesis [38,39]. Therefore, RipE1 or another effector(s) in the 11 effectors deleted in the undecuple mutant might suppress defense responses other than PTI, for example, ETI, in resistant plants. It would be interesting to determine whether any of the 11 effectors show the ability to affect ETI in *N. benthamiana*.

## 4. Materials and Methods

### 4.1. Plant, Yeast, and Bacterial Growth Conditions

*N. benthamiana*, *N. sylvestris*, and *A. thaliana* were grown in a controlled environment room, as described previously [21]. The bacterial strains used in this study are listed in Table S1. The growth conditions, media, and antibiotics used for the *Escherichia coli*, *Saccharomyces cerevisiae*, *R. solanacearum*, Pto, and *A. tumefaciens* strains were described previously [19,21].

### 4.2. Agrobacterium-Mediated Transient Expression (Agroinfiltration)

*ripE1* and *JAZs* were cloned into the *Nco*I-*Eco*RV sites of the pENTER4 vector (Invitrogen, Carlsbad, CA, USA) using an In-Fusion HD cloning kit. *ripE1^C172A^* and *ripE1^H203A^* were produced by PCR-based site-directed mutagenesis. The resultant entry clones were subcloned into pGWB5 and pGWB15 vectors [40] using a Gateway LR Clonase II enzyme mix (Invitrogen). *A. tumefaciens* cells harboring the resultant plasmids were suspended in infiltration buffer [10 mM MgCl_2_, 10 mM MES (pH 5.6)] supplemented with 150 μM acetosyringone. The inoculum preparations were spectrometrically adjusted to OD_600_ = 1.0 and incubated at 30 °C for 3 h with shaking before infiltration. For coexpression assays, *A. tumefaciens* cells harboring pDGBα2-35S:P19 [41], pGWB5 derivatives, and pGWB15 derivatives were mixed at a ratio of 1:3:3 and infiltrated into the leaves of *N. benthamiana*. The primer sets used for plasmid construction are listed in Table S2.

### 4.3. Measurement of ROS

ROS were measured using a chemiluminescence probe L-012 (Wako, Osaka, Japan) as described previously [21]. Leaf disks were floated on water overnight. Then, the water was replaced with 0.5 mM L-012 solution (10 mM MOPS-KOH, pH 7.4) containing 100 nM flg22 (Funakoshi, Tokyo, Japan). Chemiluminescence was continuously monitored using a microplate reader (SH-8000Lab, Corona Electric, Ibaraki, Japan).

### 4.4. Protein Extraction and Immunoblotting

Leaf disks (60 mg) were collected, frozen in liquid nitrogen, and ground to a fine powder. Proteins were extracted in 100 μL of extraction buffer (0.35 M Tris-HCl (pH 6.8), 30% glycerol, 10% SDS, 0.6 M DTT, 0.012% bromophenol blue). The fraction of nuclear proteins was extracted from the leaves using a CelLytic PN Isolation/Extraction kit (Sigma–Aldrich, St. Louis, MO, USA) in accordance with the manufacturer’s instructions. Total protein (5 μL) was separated by 10% SDS-PAGE. Separated proteins were transferred onto a membrane and incubated with an HRP-conjugated anti-GFP (1:5000; Miltenyi Biotec, Bergisch Gladbach, Germany), anti-HA (1:2000; MBL, Aichi, Japan, #561-7), anti-RFP (1:2000; MBL, #PM005-7), anti-GAPDH (1:5000; Proteintech, Rosemont, IL, USA), or anti-histone H3 (1:2000; MBL) antibody. Immunodetection was performed using an ECL Prime Western blotting detection reagent (GE Healthcare, Marlborough, MA, USA) or Clarity Max Western ECL Substrate (Bio-rad, Hercules, CA, USA).

### 4.5. Protease Activity Assays

Protease activity assays were performed as described previously [27] with slight modifications. Briefly, leaves of *N. benthamiana* plants transiently expressing HA-tagged RipE1, RipE1^C172A^, and RipE1^H203A^ were homogenized in an extraction buffer containing 100 mM Tris-HCl (pH 7.5), 150 mM NaCl, 5 mM EDTA, 5% glycerol, 10 mM DTT, 1 mM PMSF, 0.5% Triton X-100, and a protease inhibitor cocktail (Roche, Mannheim, Germany). HA-tagged proteins were purified from the homogenate using an anti-HA affinity resin (Pierce, Rockford, IL, USA). Protease activity was determined using a Protease Fluorescent Detection kit (Sigma–Aldrich) in accordance with the manufacturer’s instructions. Fluorescence intensity was measured at 485 nm excitation and 535 nm emission using a microplate reader.

### 4.6. Measurement of Ion Leakage and Chlorophyll Content

The severity of cell death was quantified by the degree of electrolyte leakage from leaves. Leaf disks (8 mm in diameter) were immersed in 1 mL of water for 2 h with gentle shaking. The ion conductivity of water was measured using a conductivity meter (LAQUAtwin, Horiba, Kyoto, Japan).

The chlorophyll content of the leaves was spectrophotometrically measured using a chlorophyll meter (SPAD-502, Konica Minolta, Tokyo, Japan) in accordance with the manufacturer’s instructions.

### 4.7. Real-Time PCR Analysis

Real-time PCR was performed as described previously [42]. Briefly, total RNA was extracted from leaves using an RNeasy Plant Mini Kit (Qiagen, Hilden, Germany), and cDNA was synthesized using a High Capacity cDNA reverse transcription kit (Applied Biosystems, Foster, CA, USA). Quantitative PCR was performed using a Power SYBR Green PCR master mix (Applied Biosystems). Expression levels of target genes were normalized to those of multiple endogenous control genes, such as *NbEF1α*, *NbNQO*, and *NbF-box*. Gene-specific primer sets used for the real-time PCR analysis are listed in Table S3.

### 4.8. Microscopic Analyses

*ripE1* and its derivative genes were cloned into the *Bam*HI site of the SUPERR:sXVE:GFP_N_:Hyg vector [43] using an In-Fusion HD cloning kit. The resultant plasmids were used for *Agrobacterium*-mediated transient expression in *N. benthamiana* leaves. *A. tumefaciens* cells harboring SUPERR:sXVE:mCherry_N_:Bar vector were coinfiltrated and used as a nucleocytoplasm marker. Leaves were treated with 50 μM β-estradiol (Wako) 1 d after the agroinfiltration. The fluorescence of GFP and RFP was observed 1 day after the treatment with β-estradiol under a laser scanning microscope (FV1200, Olympus, Tokyo, Japan).

For BiFC analysis, the entry clones of *ripE1* and its derivatives and *JAZs* were, respectively, subcloned into the pB5nYGW and pB5GWcY vectors [44] using a Gateway LR Clonase II enzyme mix. *A. tumefaciens* cells harboring pDGBα2-35S:P19, pB5nYGW derivatives, and pB5GWcY derivatives were mixed at a ratio of 1:3:3 and infiltrated into the leaves of *N. benthamiana*.

### 4.9. Yeast Two-Hybrid Analysis

Yeast two-hybrid analysis was performed using the Matchmaker Gold yeast two-hybrid system (Clontech, Palo Alto, CA, USA) in accordance with the manufacturer’s instructions. *ripE1* and its derivatives were cloned into the *Eco*RI-*Bam*HI sites of the pGBKT7 vector and transformed into *S. cerevisiae* Y2HGold as the bait. For the identification of RipE1 targets, yeast two-hybrid screening was performed using Make Your Own Mate & Plate Library System (Clontech) in accordance with the manufacturer’s instructions. Briefly, total RNA was extracted from *A. thaliana* leaves infiltrated with *R. solanacearum* RS1002 (1 × 10^8^ CFU mL^−1^) and 1 μM flg22. A mixture of total RNA was used to synthesize the cDNA library and ligated into the pGADT7-Rec vector. The resultant plasmids were transformed into *S. cerevisiae* Y187 as prey. Positive clones were selected on QDO (SD/−Ade/−His/−Leu/−Trp) stringent selective plates by mating the Y2HGold and Y187 strains.

For the validation of RipE1 and JAZs interaction, the entry clones for *JAZs* were obtained from ABRC and RIKEN BRC through the National Bio-Resource Project of MEXT/AMED, Japan. The entry clones were subcloned into the pGADT7-GW vector [45] using a Gateway LR Clonase II enzyme mix. The resultant plasmids were transformed into Y187. Diploid cells harboring pGBKT7 and pGADT7 derivatives were selected on DDO (SD/−Leu/−Trp) plates. Positive interactions of RipE1s and JAZs were tested on TDO (SD/−His/−Leu/−Trp) moderate selective plates and QDO plates.

### 4.10. Generation of R. solanacearum Mutants

Each fragment (0.6 kb) upstream and downstream of the effector coding region was tandemly inserted into the plasmid pK18*mobsacB* [46] using an In-Fusion HD cloning kit (Takara, Shiga, Japan) The resultant plasmids were used to generate *R. solanacearum* mutants by the marker-exchange method using *E. coli* S17-1 [46].

### 4.11. Bacterial Virulence Assay

The virulence of *R. solanacearum* and Pto in plants was assayed, as described previously [21]. For measuring the growth of *R. solanacearum* strains, the inoculum (1 × 10^4^ CFU mL^−1^) was infiltrated into the leaves of *Nicotiana* plants using a needleless syringe. For measuring the growth of Pto, *A. thaliana* was treated with 100 μM β-estradiol supplemented with 0.01% Silwet L-77 for 1 day and then inoculated by spraying the inoculum (1 × 10^8^ CFU mL^−1^) containing 10 mM MgCl_2_ and 0.01% Silwet L-77. Leaf disks were taken from the inoculated leaves and homogenized in water. Serial dilutions of the homogenate were spread on BG plates containing nalidixic acid for *R. solanacearum* and King’s B plates containing rifampicin for Pto.

## Supplementary Materials

Supplementary materials can be found at https://www.mdpi.com/1422-0067/20/23/5992/s1.

## Abbreviations

PTI	pattern-triggered immunity
PAMPs	pathogen/microbe-associated molecular patterns
PRRs	pattern-recognition receptors
ROS	reactive oxygen species
SA	salicylic acid
JA	jasmonic acid
COR	coronatine
Pto	*Pseudomonas syringae* pv. *tomato*
COI1	Coronatine-insensitive1
JAZ	Jasmonate-ZIM-domain
ETI	effector-triggered immunity
HA	hemagglutinin
GFP	green fluorescent protein
BiFC	bimolecular fluorescence complementation
