# Supplementary material for: Comprehensive Identification of PTI Suppressors in Type III Effector Repertoire Reveals that Ralstonia solanacearum Activates Jasmonate Signaling at Two Different Steps
Source: Int J Mol Sci. 2019 Nov 28;20(23):5992. doi: 10.3390/ijms20235992 (PMC6928842; doi:10.3390/ijms20235992)
Supplement: Supplementary file 1 [file ijms-20-05992-s001.zip › Supplemental Materials/Supplemental Figures.pdf]

```
RipE1     10   RCFRPAVSRPEAETAAPSSSQEHDRPGSPERSPRRAPAALQGLTPRAESSRRQAAPEAPA   69
HopX1Pta  8    HSLPAPGPSVETTEKAVQSSSAQNPASCSSQTERPEAGSTQ-VRPNYPYSSVKTRLPPVS   66

RipE1     70   GPARFLTDGERQFGGYLMARDVDQRPVHGEPLDTLRSANETLLQTRRILTHGRGNVEDDI   129
HopX1Pta  67   STGQAISDTPSSLPGYLLLRRLDRRPLDEDSIKALVPADEAVREARRALPFGRGNIDVDA   126

                                                                  *
RipE1     130  DATHGLSTHIAQGGRSI-QESMWRAH-PKP----VVWAA-IAM---VAGAGNCGEHADLA   179
HopX1Pta  127  QRTHLQSGARAVAAKRLRKDAERAGHEPMPGNDEMNWHVLVAMSGQVFGAGNCGEHARIA   186

                                        *                 *
RipE1     180  TFLHAAKLKE--GEAVDNVHI---DDFDHFWAIVHRAEPDLERDVYIDAWGKGPAIFAVD   234
HopX1Pta  187  SFAYGALAQESGRSPREKIHLAEQPGKDHVWAETDNSSAG-SSPIVMDPWSNGAAILAED   245

RipE1     235  GMMTYRPGERRTKFGYDKA-SGEEAHADMEMLATVL---ATRMRGGISNTMHRLGP--DY   288
HopX1Pta  246  SRFAKDRSAVERTYSFTLAMAAEAGKVTRETAENVLTHTTSRLQKRLADQLPNVSPLEGG   305

RipE1     289  RYPPERVWAVTPIVAQRFTDRVKAEMSKPADLGKLAVPPDCATPSSVEPPVTNERLMQPL   348
HopX1Pta  306  RYQQEKS-VLDEAFARRVSDKLNSDDPRRA-LQMEIEAVGVAMSLGAEGVKTVARQAPKV   363

RipE1     349  RHEIHATRIARTLGAH   364
HopX1Pta  364  VRQARSVASSKGMPPR   379
```

Figure S1. Amino acid sequences of RipE1 and HopX1$_{Pta}$. Protein sequences were aligned using the GENETYX program. Identical and highly conserved amino acid residues are shown in black and gray, respectively. Asterisks (*) denote the putative catalytic residues required for protease activity. The positions of amino acid residues are indicated by numbers. Accession numbers: RipE1 (AB302270) from *R. solanacearum* and HopX1$_{Pta}$ (ACR46710) from *P. syringae* pv. *tabaci*.

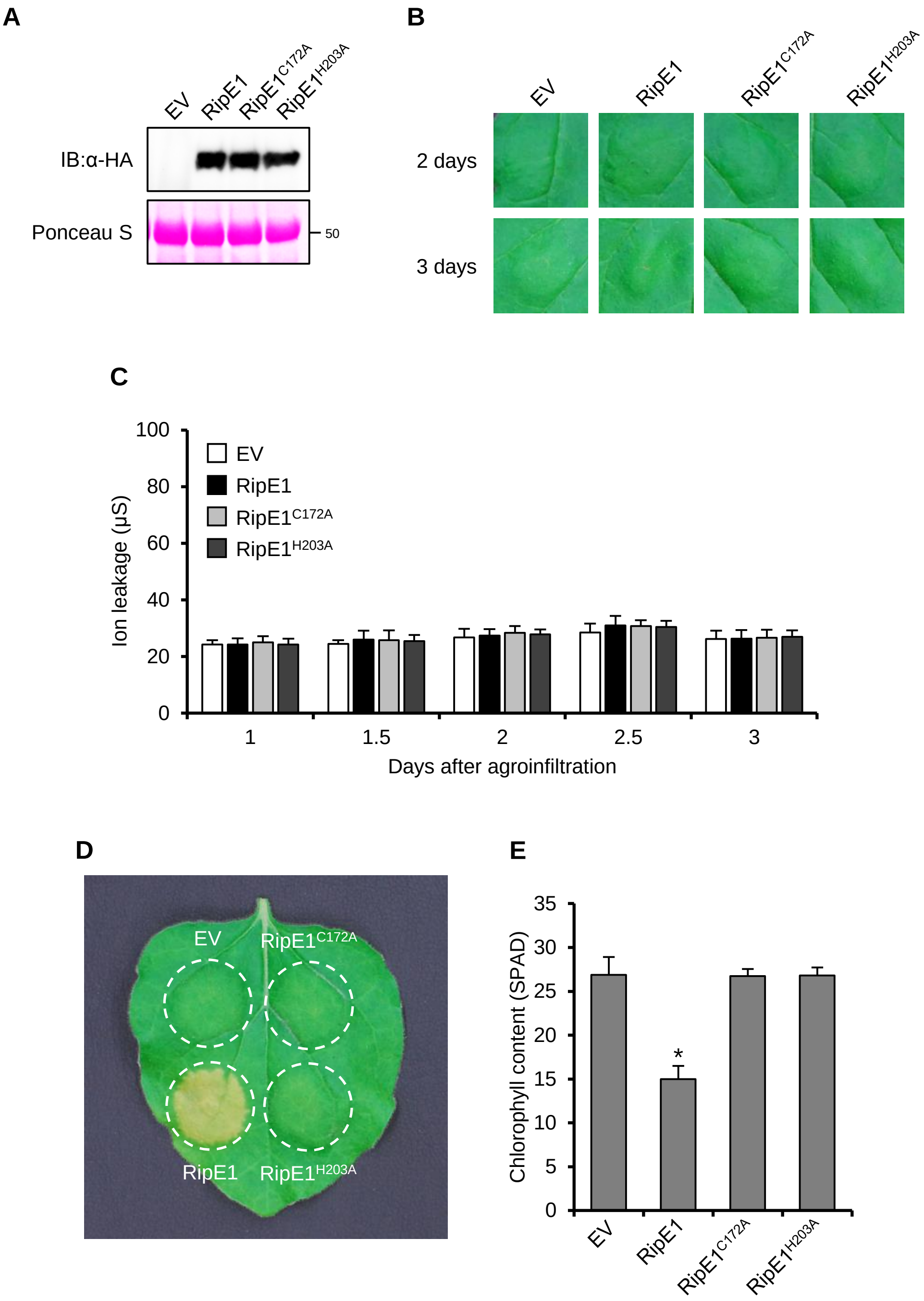

Figure S2. Transient expression of RipE1 in *N. benthamiana*. Leaves were infiltrated with *A. tumefaciens* harboring the binary vector expressing N-terminal HA-tagged RipE1, RipE1^{C172A}, or RipE1^{H203A}, or EV. (A) Immunoblot analysis of RipE1 and its putative catalytic site mutants. Total protein was extracted from leaves 2 days after agroinfiltration and subjected to immunoblot analysis using an anti-HA antibody. The membrane was stained with Ponceau S as the loading control. (B) Morphological features of leaves expressing RipE1 and its mutants. Photographs were taken 2 and 3 days after agroinfiltration. (C) Level of ion leakage in (B). The degree of ion leakage from leaves was measured on the indicated days after agroinfiltration. Values are means ±SD of six replicates. (D) Chlorotic symptom of leaves expressing RipE1 and its mutants. Photographs were taken 1 week after agroinfiltration. (E) Chlorophyll content in (D). Foliar chlorophyll content was measured using a chlorophyll meter. Values are means ±SD of six replicates. The asterisk (*) denotes a statistically significant difference compared with the EV control ($P<0.01$, Student's t-test). All experiments were repeated three times with similar results, and representative results are shown.

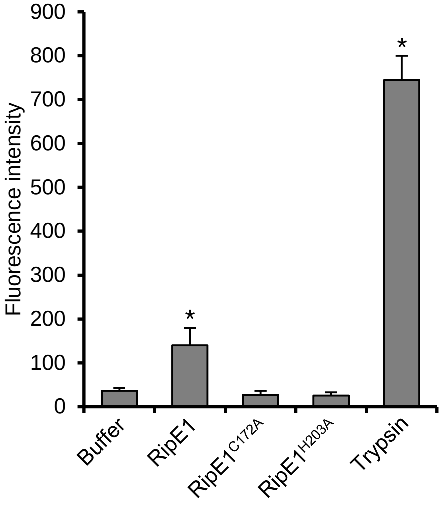

Figure S3. Protease activity of RipE1 and its putative catalytic site mutants in vitro. Leaves of *N. benthamiana* were infiltrated with *A. tumefaciens* harboring the binary vector expressing N-terminal HA-tagged RipE1, RipE1^{C172A}, or RipE1^{H203A}. Recombinant HA-tagged proteins were incubated with the fluorescein-labeled casein substrate. Assay buffer and trypsin solution were used as the negative and positive controls, respectively. Asterisks (*) denote statistically significant differences compared with the negative control (P<0.01, Student's t-test).

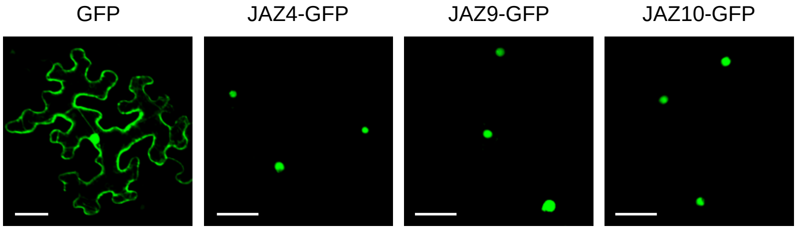

Figure S4. Subcellular localization of JAZs in *N. benthamiana* cells. Leaves were infiltrated with *A. tumefaciens* harboring the binary vector expressing C-terminal GFP-tagged JAZ4, JAZ9, or JAZ10, or GFP alone. Fluorescence was observed 2 days after agroinfiltration by confocal microscopy. Bars, 50 μm.

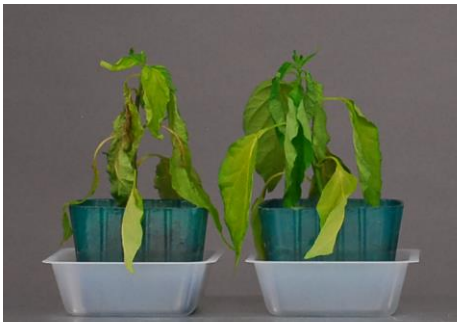

Figure S5. Disease symptoms in *Capsicum annuum* inoculated with *R. solanacearum* strains. Stems were inoculated with the suspension of the wild-type or Δ*ripE1* strain. Photographs were taken 7 days after inoculation.

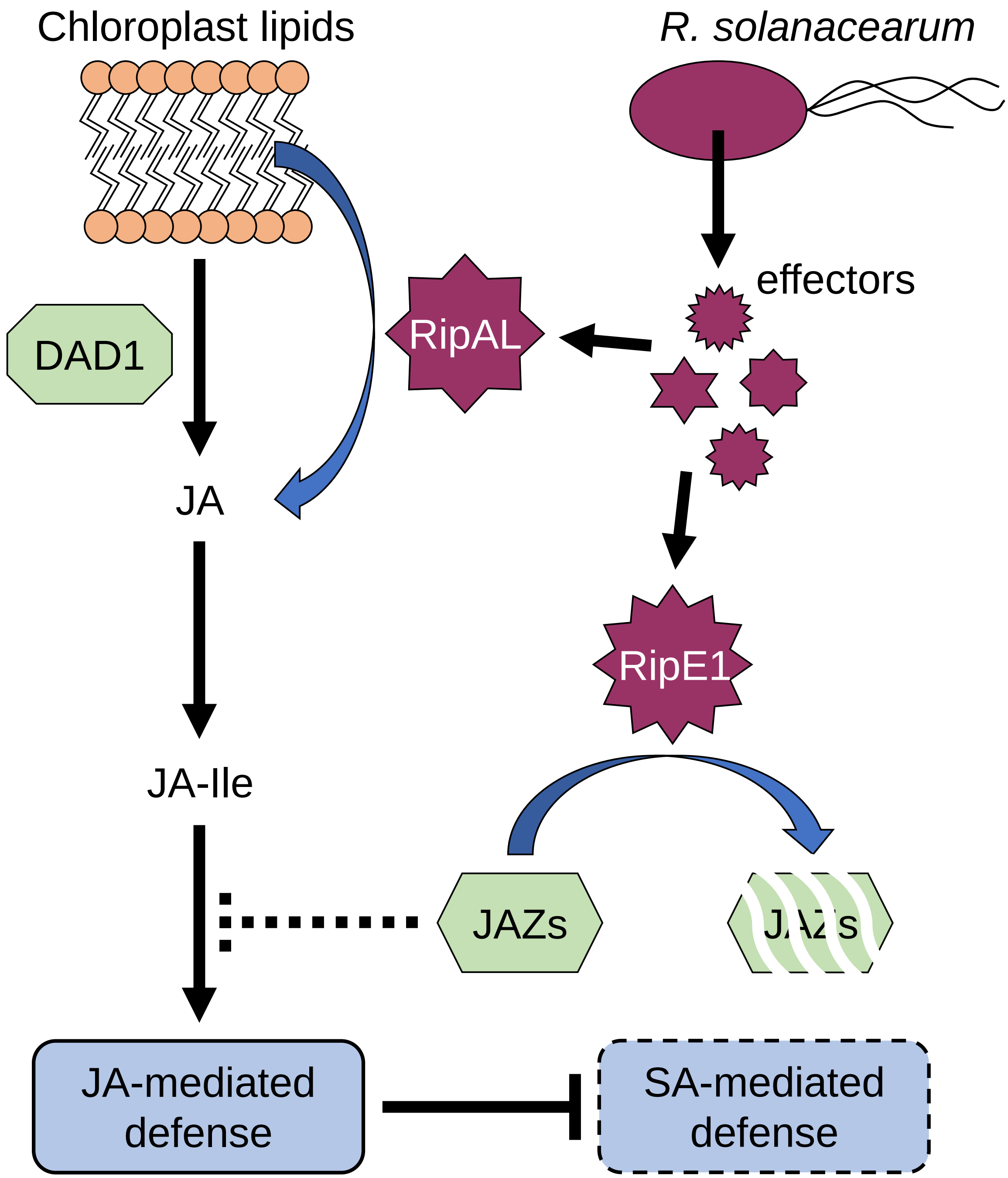

Figure S6. Proposed model for the functions of *R. solanacearum* RipE1 and RipAL effectors in the activation of host JA signaling. The putative lipase effector RipAL releases JA precursors from chloroplast lipids and induces JA production. The increase in JA and JA-Ile levels activates the upstream of the JA signaling pathway. The cysteine protease effector RipE1 degrades JAZ repressors and activates the downstream of the JA signaling pathway. The activations of JA signaling at two different steps strongly suppress the antagonistic SA signaling that is required for the defense response against *R. solanacearum*.
