# Supplementary material for: Comprehensive Identification of PTI Suppressors in Type III Effector Repertoire Reveals that Ralstonia solanacearum Activates Jasmonate Signaling at Two Different Steps
Source: Int J Mol Sci. 2019 Nov 28;20(23):5992. doi: 10.3390/ijms20235992 (PMC6928842; doi:10.3390/ijms20235992)
Supplement: Supplementary file 1 [file ijms-20-05992-s001.zip › Supplemental Materials/Table_S1.pdf]

TABLE S1 Bacterial and yeast strains used in this study.

Strain	Relevant genotype	Reference
Escherichia coli		
S17-1	*E. coli* K12 *thi pro hsdR*$^{-}$ *hsdM*$^{+}$ *recA* [chr::RP4-2-Tc::Mu-Km::Tn*7*]	[1]
Saccharomyces cerevisiae		
Y2HGold	*MATa*, *trp1-901*, *leu2-3*, *112*, *ura3-52*, *his3-200*, *gal4*Δ, *gal80*Δ, *LYS2*::*GAL1*$_{UAS}$–*Gal1*$_{TATA}$–*His3*, *GAL2*$_{UAS}$–*Gal2*$_{TATA}$–*Ade2* *URA3*::*MEL1*$_{UAS}$–*Mel1*$_{TATA}$ *AUR1-C MEL1*	Clontech
Y187	*MATα*, *ura3-52*, *his3-200*, *ade2-101*, *trp1-901*, *leu2-3*, *112*, *gal4*Δ, *gal80*Δ, *met–*, *URA3*::*GAL1*$_{UAS}$–*Gal1*$_{TATA}$–*LacZ*, *MEL1*	[2]
Ralstonia solanacearum		
RS1002	RS1000 Nal^{r} (phylotype I, biovar 4)	[3]
RS1704	RS1002 Δ*ripAW* Δ*ripAR*	[4]
RS1720	RS1002 Δ*ripE1*	This study
RS1725	RS1002 Δ*ripAR* Δ*ripAW* Δ*ripAY*	This study
RS1726	RS1002 Δ*ripAL* Δ*ripAR* Δ*ripAW* Δ*ripAY*	This study
RS1727	RS1002 Δ*ripE1* Δ*ripAL* Δ*ripAR* Δ*ripAW* Δ*ripAY*	This study
RS1728	RS1002 Δ*ripE1* Δ*ripAC* Δ*ripAL* Δ*ripAR* Δ*ripAW* Δ*ripAY*	This study
RS1729	RS1002 Δ*ripE1* Δ*ripAC* Δ*ripAL* Δ*ripAP* Δ*ripAR* Δ*ripAW* Δ*ripAY*	This study
RS1730	RS1002 Δ*ripE1* Δ*ripAC* Δ*ripAL* Δ*ripAP* Δ*ripAR* Δ*ripAU* Δ*ripAW* Δ*ripAY*	This study
RS1731	RS1002 Δ*ripE1* Δ*ripQ* Δ*ripAC* Δ*ripAL* Δ*ripAP* Δ*ripAR* Δ*ripAU* Δ*ripAW* Δ*ripAY*	This study
RS1732	RS1002 Δ*ripA5* Δ*ripE1* Δ*ripQ* Δ*ripAC* Δ*ripAL* Δ*ripAP* Δ*ripAR* Δ*ripAU* Δ*ripAW* Δ*ripAY*	This study
RS1733	RS1002 Δ*ripA5* Δ*ripE1* Δ*ripI* Δ*ripQ* Δ*ripAC* Δ*ripAL* Δ*ripAP* Δ*ripAR* Δ*ripAU* Δ*ripAW* Δ*ripAY*	This study
Pseudomonas syringae pv. *tomato*		
DC3000	Rif^{r} COR^{+}	[5]
DC3000 *cor*$^{-}$	DC3000 Δ*cmaA* Δ*cfa6* (Rif^{r} COR^{-})	[6]
Agrobacterium tumefaciens		
GV3101	Gm^{r} Rif^{r}	[7]

Supplementary References

1. Simon, R.; Priefer, U.; Pühler, A. A broad host range mobilization system for *in vivo* genetic engineering: Transposon mutagenesis in Gram-negative bacteria. *Nature Biotechnology*. 1983, *1*, 784–791.
2. Harper, J.W.; Adami, G.R.; Wei, N.; Keyomarsi, K.; Elledge, S.J. The p21 Cdk-interacting protein Cip1 is a potent inhibitor of G1 cyclin-dependent kinases. *Cell.* 1993, *75*(4), 805–816.
3. Mukaihara, T.; Tamura, N.; Murata, Y.; Iwabuchi, M. Genetic screening of Hrp type III-related pathogenicity genes controlled by the HrpB transcriptional activator in *Ralstonia solanacearum. Mol Microbiol.* 2004, *54*(4), 863–875.
4. Nakano, M.; Oda, K.; Mukaihara, T. *Ralstonia solanacearum* novel E3 ubiquitin ligase (NEL) effectors RipAW and RipAR suppress pattern-triggered immunity in plants. *Microbiology (Reading, UK)*. 2017, *163*(7), 992–1002.
5. Cuppels, D.A. Generation and characterization of Tn5 insertion mutations in *Pseudomonas syringae* pv. *tomato*. *Appl Environ Microbiol*. 1986, *51*(2), 323–327.
6. Nakano, M.; Mukaihara, T. *Ralstonia solanacearum* type III effector RipAL targets chloroplasts and induces jasmonic acid production to suppress salicylic acid-mediated defense responses in plants. *Plant Cell Physiol.* 2018, *59*(12), 2576–2589.
7. Lamblin, F.; Saladin, G.; Dehorter, B.; Cronier, D.; Grenier, E.; Lacoux, J.; Bruyant, P.; Lainé, E.; Chabbert, B.; Girault, F.; Monties, B.; Morvan, C.; David, H.; David, A. Overexpression of a heterologous sam gene encoding S-adenosylmethionine synthetase in flax (*Linum usitatissimum*) cells: Consequences on methylation of lignin precursors and pectins. *Physiol Plant.* 2001, *112*(2), 223–232.
