# Supplementary material for: Comprehensive Identification of PTI Suppressors in Type III Effector Repertoire Reveals that Ralstonia solanacearum Activates Jasmonate Signaling at Two Different Steps
Source: Int J Mol Sci. 2019 Nov 28;20(23):5992. doi: 10.3390/ijms20235992 (PMC6928842; doi:10.3390/ijms20235992)
Supplement: Supplementary file 1 [file ijms-20-05992-s001.zip › Supplemental Materials/Table_S2.pdf]

TABLE S2 Primer sets used for vector construction.

Target gene	Purpose	Sequence (5'-3')
ripE1	Cloning	GCAGGCTCCACCATGCCGCCCGTCCTGCCGTCGAT
		AAGCTGGGTCTAGATAGCTTTCCGTGGCGGGCGGC
ripE1^{C172A}	Mutagenesis	GTGGCAGGGGCGGGGAACGCCGGCGAACACGCCGATCTG
		CAGATCGGCGTGTTCGCCGGCGTTCCCCGCCCCTGCCAC
ripE1^{H203A}	Mutagenesis	CATATCGACGACTTCGACGCCTTCTGGGCAATCGTGCAC
		GTGCACGATTGCCCAGAAGGCGTCGAAGTCGTCGATATG
ripE1 / *ripE1*^{C172A} / *ripE1*^{H203A}	Localization / Transgenic plant	GCCTACTAGTGGATCCATGCCGCCCGTCCTGCCGTC
		CGAGGTCGACGGATCCTCAGCTTTCCGTGGCGGGCGG
ripE1 / *ripE1*^{C172A} / *ripE1*^{H203A}	Yeast two-hybrid	CATGGAGGCCGAATTCATGCCGCCCGTCCTGCCGTC
		GCAGGTCGACGGATCCTCAGCTTTCCGTGGCGGGCG
JAZ4	Cloning	GCAGGCTCCACCATGGAGAGAGATTTTCTC
		AAGCTGGGTCTAGATAGTGCAGATGATGAG
JAZ9	Cloning	GCAGGCTCCACCATGGAAAGAGATTTTCTG
		AAGCTGGGTCTAGATATGTAGGAGAAGTAG
JAZ10	Cloning	GCAGGCTCCACCATGTCGAAAGCTACCATA
		AAGCTGGGTCTAGATAGGCCGATGTCGGAT
ripA5 upstream	Deletion mutant	CGACTCTAGAGGATCCGATACATGGACCGCATCCG
		GCGTTTGCTGCATCGGCTCCGGTGCACGCGCGTCG
ripA5 downstream	Deletion mutant	CGCGTGCACCGGAGCCGATGCAGCAAACGCGCCCG
		CGGTACCCGGGGATCATCCGGGTGCTGGGGTGCCT
ripE1 upstream	Deletion mutant	CGACTCTAGAGGATCCGACGATGTCCCAGACCCTC
		GAGCGCGGACGACCGGGCAGGCGGCTCAGTGTGGG
ripE1 downstream	Deletion mutant	ACTGAGCCGCCTGCCCGGTCGTCCGCGCTCAGCTC
		CGGTACCCGGGGATCCAGCTCGGAGATCCAGGTGC
ripI upstream	Deletion mutant	CGACTCTAGAGGATCCTGACCGGCTATGCCAGCATC
		CGGCCTTTCAGGTCGCCGTACACCTATGTCATATTCGG
ripI downstream	Deletion mutant	GACATAGGTGTACGGCGACCTGAAAGGCCGGCCTG
		CGGTACCCGGGGATCCCTCACCAAGCAGTACCAGG
ripQ upstream	Deletion mutant	CGACTCTAGAGGATCCTCCAAGGGGCAGGAGTGGA
		CGCCGGCTTTGCGCGGATTCGGATTCCGTTTGCGG
ripQ downstream	Deletion mutant	AACGGAATCCGAATCCGCGCAAAGCCGGCGAGCAG
		CGGTACCCGGGGATCTGGCAGCCAAGCCGCTGGGC
ripAC upstream	Deletion mutant	CCCAAGCTTACGCAGCCGATGTAATGGCG
		GCTCTAGACCGAACGATCAGTCGTCGTC

ripAC downstream	Deletion mutant	CGGGATCCTCCGGTGCCGCATCCCCGCC
		GGAATTCCGCTACCTGATGGCGCGCAT
ripAL upstream	Deletion mutant	GGAAGCTTTTTCCCGATGCCGCAGCGTTC
		GGGGATCCGGGGCGGTCAAAGCAGCATCGG
ripAL downstream	Deletion mutant	GGGGATCCGTCGCAGGTGGCAGGCCAACGG
		GGGAATTCCGGATTGGGGATTGAGGACGAC
ripAP upstream	Deletion mutant	CGACTCTAGAGGATCCGCGGGCGTGCTCCTTGGCC
		CAGCGAGCCCGACGGGATGACGCGACGGTGCTGGG
ripAP downstream	Deletion mutant	CACCGTCGCGTCATCCCGTCGGGCTCGCTGTTGCG
		CGGTACCCGGGGATCCTGGCCGCCTCCACCAGCTTG
ripAU upstream	Deletion mutant	CGACTCTAGAGGATCTGGATCGACGACACTGTATC
		CAACCGGCGTGCCCGCGGAAACGTGTGGGGTTGCA
ripAU downstream	Deletion mutant	CCCCACACGTTTCCGCGGGCACGCCGGTTGACGCG
		CGGTACCCGGGGATCGGTACAGCGCCTCGTACGCG
ripAY upstream	Deletion mutant	GGGAATTCCGGCCCGCCATGCGG
		GGGGATCCGGCTGGCACCGTCGATCGGGA
ripAY downstream	Deletion mutant	GGGGATCCCGCCACTCGCCATTCTCTCGC
		GGAAGCTTAGCCGATGCCGATAATGTCC
