# Supplementary material for: Comprehensive Identification of PTI Suppressors in Type III Effector Repertoire Reveals that Ralstonia solanacearum Activates Jasmonate Signaling at Two Different Steps
Source: Int J Mol Sci. 2019 Nov 28;20(23):5992. doi: 10.3390/ijms20235992 (PMC6928842; doi:10.3390/ijms20235992)
Supplement: Supplementary file 1 [file ijms-20-05992-s001.zip › Supplemental Materials/Table_S3.pdf]

TABLE S3 Primer sets used for real-time PCR analysis.

Target gene	Purpose	Sequence (5'-3')	Reference
NbCyp71D20	qRT-PCR	TCATGGCGACCACTGGAGACAAAT	[1]
		TGATGGAGCTGAAGGACCGAACAT	
NbAcre31	qRT-PCR	GAGAAACTGGGATTGCCTGAAGGA	[2]
		AATTCGGCCATCGTGATCTTGGTC	
NbGras2	qRT-PCR	TCATGAGGCGTTACTCGGAGCATT	[2]
		TACCTAGCACCAAGCAGATGCAGA	
NbWrky22	qRT-PCR	CAAGGCATAAAGCAGACACAACA	[1]
		GCTTCTGACCACCGCATGT	
NbAOS	qRT-PCR	TCCGTTAGACACCAATCTTGGA	[3]
		AGGCCTAAAATTAGCAAAGGATGA	
NbPR4	qRT-PCR	GGCCAAGATTCCTGTGGTAGAT	[4]
		CACTGTTGTTTGAGTTCCTGTTCCT	
NbICS1	qRT-PCR	GTGTCGGCTCTGCTGTCTTCT	[5]
		CTGCGTATAGCACGCCAATC	
NbEF1α	qRT-PCR	CCCAAGAGGCCCTCAGACA	[1]
		CACACGACCAACAGGGACAGT	
NbNQO	qRT-PCR	AAGGCGGTGGTCAAGAAA	[6]
		CAAACATACCAGCACCGAATG	
NbF-box	qRT-PCR	GGCACTCACAAACGTCTATTTC	[7]
		ACCTGGGAGGCATCCTGCTTAT	

Supplementary References

1. Heese, A.; Hann, D.R.; Gimenez-Ibanez, S.; Jones, A.M.; He, K.; Li, J.; Schroeder, J.I.; Peck, S.C.; Rathjen, J.P. The receptor-like kinase SERK3/BAK1 is a central regulator of innate immunity in plants. *Proc Natl Acad Sci U S A*. 2007, *104*(29), 12217–12222.
2. Nguyen, H.P.; Chakravarthy, S.; Velasquez, A.C.; McLane, H.L.; Zeng, L.; Nakayashiki, H.; Park, D.H.; Collmer, A.; Martin, G.B. Methods to study PAMP-triggered immunity using tomato and *Nicotiana benthamiana*. *Mol Plant Microbe Interact.* 2010, *23*(8), 991–999.
3. Chung, H.S.; Koo, A.J.; Gao, X.; Jayanty, S.; Thines, B.; Jones, A.D.; Howe, G.A. Regulation and function of Arabidopsis *JASMONATE ZIM*-domain genes in response to wounding and herbivory. *Plant Physiol*. 2008, *146*(3), 952–64.
4. Maimbo, M.; Ohnishi, K.; Hikichi, Y.; Yoshioka, H.; Kiba, A. Induction of a small heat shock protein and its functional roles in *Nicotiana* plants in the defense response against *Ralstonia solanacearum. Plant Physiol.* 2007, *145*(4), 1588–1599.
5. Nakano, M.; Mukaihara, T. *Ralstonia solanacearum* type III effector RipAL targets chloroplasts and induces jasmonic acid production to suppress salicylic acid-mediated defense responses in plants. *Plant Cell Physiol.* 2018, *59*(12), 2576–2589.
6. Pombo, M.A.; Ramos, R.N.; Zheng, Y.; Fei, Z.; Martin, G.B.; Rosli, H.G. Transcriptome-based identification and validation of reference genes for plant-bacteria interaction studies using *Nicotiana benthamiana. Sci Rep.* 2019, *9*(1), 1632.
7. Liu, D.; Shi, L.; Han, C.; Yu, J.; Li, D.; Zhang, Y. Validation of reference genes for gene expression studies in virus-infected *Nicotiana benthamiana* using quantitative real-time PCR. *PLoS One.* 2012, *7*(9), e46451.
